# One Does Not Fit All: European Study Shows Significant Differences in Value-Priorities in Clean Sport

**DOI:** 10.3389/fspor.2021.662542

**Published:** 2021-05-24

**Authors:** Toby Woolway, Anne-Marie Elbe, Vassilis Barkoukis, Kevin Bingham, Konstantin Bochaver, Dmitriy Bondarev, Andy Hudson, Lara Kronenberg, Lambros Lazuras, Luca Mallia, Yannis Ntovolis, Arnaldo Zelli, Andrea Petróczi

**Affiliations:** ^1^School of Life Sciences, Pharmacy and Chemistry, Kingston University London, London, United Kingdom; ^2^Institute for Sport Psychology and Sport Pedagogy, Leipzig University, Leipzig, Germany; ^3^Department of Physical Education and Sport Science, Aristotle University of Thessaloniki, Thessaloniki, Greece; ^4^Centre for Behavioural Science and Applied Psychology, Sheffield Hallam University, Sheffield, United Kingdom; ^5^Laboratory of Sport Psychology, Moscow Institute of Psychoanalysis, Moscow, Russia; ^6^Institute of Living Systems, University of Jyväskylä, Jyväskylä, Finland; ^7^Faculty of Sport and Health Sciences, Immanuel Kant Baltic Federal University, Kaliningrad, Russia; ^8^Department of Movement, Human and Health Science, University of Rome “Foro Italico”, Rome, Italy; ^9^Department of Movement Sciences, Katholieke Universiteit Leuven, Leuven, Belgium

**Keywords:** sport values, culture, clean sport, values-based education, anti-doping

## Abstract

Doping violates the Spirit of Sport and is thought to contradict the values which underpin this spirit. Values-based education (VBE) has been cited as a key element for creating a clean sport culture across age groups. Culturally relevant VBE requires understanding of the values that motivate athletes from different countries to practice their sport and uphold clean sport values. WADA's new International Standards for Education makes this study both needed and timely. Overall, 1,225 athletes from Germany, Greece, Italy, Russia, and the UK responded to measures assessing their general values, Spirit of Sport values, and their perceived importance of “clean sport”. MaxDiff analysis identified the most important values to participants based on their respective country of residence. Correlation analysis was conducted to assess the relationship between importance of clean sport and Spirit of Sport values. There were significant differences between participant nationality and their perceived importance of clean sport [*F*_(4, 1,204)_ = 797.060, *p* < 0.000], the most important general values (*p* < 0.05), and Spirit of Sport values (*p* < 0.05). Moderate positive correlations were observed between the perceived importance of clean sport and honesty and ethics (*r* = 0.538, *p* < 0.005) and respecting the rules of sport (*r* = 0.507, *p* < 0.005). When designing the values-based component of anti-doping education programs, athletes' different value-priorities across countries should be considered.

## Introduction

Doping represents a social, institutional, and moral problem which undermines the Spirit of Sport, and counters the values that underpin sport and anti-doping organizations globally (Donovan et al., 2002; Engelberg et al., 2015; Petróczi et al., 2017; WADA, 2021a). Since the creation of WADA in 1999, anti-doping organizations (ADOs), at national and/or regional levels, have attempted to prevent athletes' use of doping substances and methods through increased and improved doping control procedures and the use of the Whereabouts system (Gatterer et al., 2020; Woolway et al., 2020).

Clean sport is a valued and desired state that sport governing bodies, including anti-doping, strive to preserve. Athletes, spectators, and sponsors alike desire clean and fair sport, and it is this that gives legitimacy to anti-doping efforts. These shared values and priorities render the anti-doping effort ethically justifiable (Woolway et al., 2020). This shared sentiment and tacit agreement about the importance of the Spirit of Sport values justify infringements and inconvenience to athletes around doping control testing, limitation on privacy and personal freedom, and underpins anti-doping education. Gatterer et al. (2020) highlighted the need to assign a higher priority to prevention-orientated strategies, such as education and information rather than the limited detection-based deterrence strategies. In addition, group-based interventions that target the underlying causes of doping are considered to be more effective than stand-alone individual-focused programs (Petróczi et al., 2017). One such avenue of possibility for education interventions is values-based education (VBE), described by Sir Craig Reddie, former WADA President, as “one of the best weapons in the battle for clean sport.”

Aspiring to protect clean sport and its values is the cornerstone of the anti-doping movement (WADA, 2021a). The “clean” label in competitive top-level sport represents authenticity in performance and fairness in competition; therefore, it is a positive and desired attribute for fans, spectators, sponsors, and participants. Yet, precise and practical definition of what clean as a desired state or goal really means in sport is notably lacking. This is an important delimiting factor because the implicit understanding of what “clean” is determines the measures employed to protect clean sport. For example, if “clean sport” is conceptualized as “drug-free sport” (as it has been dominant in anti-doping) then efforts in countermeasures concentrate on regulation of performance-enhancing substances and detection of prohibited drugs. Acquiring a consensus on the definition of “clean” from the sports community is difficult. The academic literature is no short of debate about whether “clean sport” and its values—rooted in the values of amateur sport promoted in the Olympic Charter—is an archaic, unachievable, and impractical ideal for today's competitive sport, or if it is understood universally as “drug-free sport” (e.g., Dimeo, 2016; Englar-Carlson, 2018; Loland, 2018; Petróczi, 2021). Many argue that anti-doping needs a different approach to fighting a war on drugs (e.g., Kayser and Smith, 2008; Smith and Stewart, 2015; Mazanov and Woolf, 2017; Kayser and De Block, 2020).

Providing a uniform operational definition of “clean sport” is equally difficult. For anti-doping advocates, safeguarding clean sport values means a fight against drugs. For sport federations, it could also means tackling problems such as performance and competition manipulation, match-fixing and gambling, corruption, technological cheating, or classification manipulation. Consequently, policies put in place for protection by sport governing bodies are based on a tacit, problem-specific understanding of what “clean” is. With continued advances in sports science and sports medicine involving non-prohibited means designed to enhance training and competition, clean sport is not and cannot be equated only to “drug free” sport. Performance enhancement as such is not prohibited; in fact it is desired, encouraged, and supported as long as it is achieved through means like training, legitimate specialized equipment, special and tailored nutrition regimes, and some other aids (Petróczi, 2013; Petróczi et al., 2017). It is prohibited only if it becomes *cheating*, where performance enhancement is achieved by doping means and methods identified in the Prohibited List, which is updated yearly, and/or by a series of uncoordinated and disassociated rules and regulations across sport, if the cheating is not doping related. “Clean sport” in this sense for competitive athletes is sport where all rules that address some form of cheating in sport should be respected, and thus where equal opportunity to perform is guaranteed and winning is determined by natural abilities, effort, and desire. In essence, clean sport is cheating-free sport and clean sport behavior is an act with integrity, within the set and voluntarily accepted rules. With regard to recreational and fitness sport, clean sport could be defined as training in a healthy manner and abstaining from performance- and appearance-enhancing substances (Lazuras et al., 2017). Both angles are captured by the Spirit of Sport values, but with different priorities between competitive athletes and exercisers.

### Personal Values and Clean Sport

Values are defined as desirable, universal guiding principles that motivate actions through exerting influence on people's goal directed behavior (Schwartz, 2012). There are values which guide athletes' behavior throughout their daily lives, and there are those which are acted on by athletes within the sport environment, and underpin the Spirit of Sport (WADA, 2021a). At the individual level, values form value systems, which in turn reflect individual priorities.

Athletes who follow clean sport behavior link “clean sport” definition to fairness and rule-following (Petróczi et al., 2021). They intuitively feel that clean sport is about the timeless positive values often referred to as the Spirit of Sport, but essentially it is the moral make-up they acquired growing up where there is no room for cheating. However, the values of the spirit of amateur sport such as joy, teamwork, and playing with honesty (WADA, 2021a) may become less salient or re-constructed (Maftei et al., 2019) at the level where sport transforms to be the pursuit of better, faster, and stronger. What constitutes clean performance enhancement within the rule-following clean sport behavior, however, is highly idiosyncratic and underpinned by values important to the athletes personally (Petróczi et al., 2021). Mazanov and Huybers (2015) unearthed differences in the perceived importance of the Spirit of Sport values between the general public, amateur, and elite athletes. The latter group indicated high importance of fairness, ethics, honesty, dedication and commitment, and respect for rules and others. Mortimer et al. (2020) found that the likelihood of athletes to practice clean sport is positively predicted by moral values and the moral aspects of the Spirit of Sport. This is further supported by Ring et al. (2020) who—using hypothetical scenarios—observed that the likelihood of doping was positively and directly associated with self-enhancement values (e.g., power, achievement), but negatively and indirectly associated with self-transcendence (e.g., benevolence, universalism) and conservation (e.g., conformity, security) values. This pro-doping functional and anti-doping moral values-based dichotomy has also emerged from reasons for and against doping (Overbye et al., 2013; Erickson et al., 2015; Kegelaers et al., 2018).

Collectively, research findings linking Spirit of Sport values and doping avoidance (clean sport behavior) lead to critical important observations for the present study. Competitive athletes stay away from doping because of their values adverse to cheating (as opposed to adverse to substance use for performance enhancement). Any link between clean sport behavior and Spirit of Sport in this subpopulation therefore is likely to be caused by the close alignment of general values preventing cheating in sport with the moral values among the Spirit of Sport values.

### Value Priorities in Competitive Sport

While values themselves are relatively stable, their priorities are flexible and can differ between individuals and from one situation to another. With the broader definition of values as universal guiding principles that motivate actions through exerting influence on people's goal-directed behavior, we argue that “values as goals” (i.e., achieving a desired state) are conceptualized at the macro level but they cannot be operationalized in actual, micro level, situations. Because we are interested in how values are prioritized and structured to facilitate setting and achieving performance-related goals in competitive sport, and how this knowledge can be used to inform VBE, we regard values as general guiding principles which are contextually defined and prioritized.

Similar to the ever-changing priorities of general values at the individual level, the values underpinning the Spirit of Sport can also change to adapt to situational cues and challenges that an athlete may encounter (Woolf and Mazanov, 2017). Within the doping context, findings have indicated that an athlete's morality on doping can change over their career (Mazanov et al., 2012; Mazanov and Huybers, 2015). This was caused by a change in value priorities throughout their athletic career and as such a move from sustained abstinence to deciding to involve themselves in doping. Thus, doping, its contributing values, and the education targeting these values should take into account these situational priority changes, as well as those differences which may exist between athletes from different countries.

### Values in a Global Context

Notwithstanding the individual differences and contextual fluidity in value priorities, it is reasonable to assume that value priorities at the aggregated level are characteristics of countries and similarly to culture beliefs and are relatively stable. Nations indeed have a unique cultural profile that sets nations characteristically apart in the ways their belief and behavioral systems are endorsed and enacted at the individual level. It may be that values of sport are universal, but even if the universal and abstract values are above cultural differences, cultural differences influence the way these values are prioritized and enacted in daily practices.

Triandis (1972) defines culture as an individual's characteristic way of making sense of the world, based on the perception of rules, norms, roles, and values, influenced by various levels of culture (e.g., language, gender, race, religion, country, occupation, etc.). Based on this definition, individual reports of the norms and values are not indications of the “culture” but rather the individual's perceptions of the shared culture, which is influenced by different levels of a culture system. Intriguingly, a multi-level meta-analysis of Hofstede's cultural value dimensions (Taras et al., 2010) indicated that these trait-based cultural values were most strongly related to emotions and attitudes, particularly in culturally tight countries (Gelfand et al., 2006). Psychological adaptation of the cultural values at the individual level manifests in self-guides, self-regulation, self-monitoring abilities, and epistemic needs. Tight cultures have many strong norms and a low tolerance of deviant behavior, whereas loose cultures are characterized by having weak social norms and a high tolerance of deviant behavior (Gelfand et al., 2006, 2011). The authors propose that individuals' psychological processes become naturally attuned to, and supportive of, the situational demands in the cultural system. Individuals who are chronically exposed to stronger (vs. weaker) situations in their everyday local worlds feel that their behavioral options are limited, their actions are subject to constant evaluation, and there are potential punishments based on these evaluations. Accordingly, self-guides of individuals in nations with high situational constraint are more prevention-focused and thus are more cautious (prefer to avoid mistakes) and dutiful (focused on behaving properly) and have higher self-regulatory strength (higher impulse control), a higher need for structure, and higher self-monitoring ability. In other words, the higher (or lower) degree of social regulation that exists at the societal level is mirrored in the higher (or lower) amount of self-regulation at the individual level in tight and loose nations, respectively. Such psychological processes simultaneously reflect and support the strength of social norms and tolerance of deviance in the larger cultural context.

In social and personality psychology, theoretical and scientific attention has been devoted to the study of values and of the processes through which social values are transmitted to individuals (Caprara and Cervone, 2000). At an individual level, values stand as the criteria or standards people use to evaluate their actions, or others' actions or the benefits of behavioral alternatives (Schwartz, 1992). At a societal level, values tend to encompass the principles determining citizens' rights and duties (Brewster-Smith, 1963). Combining these two, one could say that values represent what a society conveys—through multiple socializing agencies—to all its members (Brewster-Smith, 1963). Another implication of the combination of these two levels of analysis suggests that, despite any attempt to find a small set of universal values (Schwartz, 1992), people are not simply passive recipients of social influences but, rather, they actively choose and partly shape the social factors that affect them (Caprara and Cervone, 2000).

Despite its critical importance, cross-country comparative research of values of sport (e.g., Spirit of Sport) is scarce. One study compared the importance of the Spirit of Sport values among Australian and Greek university students (Mazanov et al., 2019). The study found limited agreement about what is unimportant (“courage” and “excellence in performance”) and what is important (“ethics, fair play, and honesty” [most important for Australians] and “respect for self and others”) in sport. Other values such as “health” (top priority for Greeks), “character and education,” “community and solidarity,” or “dedication and commitment” ranked differently by the two countries. The importance of the Spirit of Sport values by British athletes appears to align with the Greeks: “health” and “respect for self and others” were ranked at the top whereas “performance excellence” and “community and solidarity” were least prioritized (Mortimer et al., 2020). However, even with the observed variation in other values, results suggest that normative anti-doping legitimacy—based on shared agreement that fair play, honesty, and authenticity in sport is worth protecting—is universal.

### VBE and WADA's International Standard for Education

The core concept of values-based anti-doping education (VBE) is to create a clean sport culture by promoting and embedding the intrinsic values of the Spirit of Sport and human integrity to empower athletes to develop reasoning and decision-making skills that protect them from doping use. In WADA's International Standard for Education (ISE; WADA, 2021b), which set out requirements for signatories, VBE is defined as “delivering activities that emphasize the development of an individual's personal values and principles. It builds the learner's capacity to make decisions to behave ethically” (p. 9). Organizations with responsibility for anti-doping [referred to as (Code) signatories] are required to develop and deliver an education program that incorporates VBE along with awareness raising, information provision about the rules, rights and responsibility, and anti-doping education focusing on specific anti-doping topics to foster clean sport behavior and ensure code compliance. The ISE also encourages signatories to consider the benefits of educating a wider population through VBE programs to promote the Spirit of Sport and broadly build a clean sport environment.

Guidelines that accompany the ISE recommend organizations to identify their local and culturally relevant values by (1) looking at the organization's vision and mission for publicly stated values, or consider setting them; and to (2) explore views of stakeholders, including athletes and athlete support personnel (ASP), and/or the general public for their opinion. It is further recommended that stakeholders could be asked to vote for a set of values including fairness, excellence, equity, respect, inclusion, fun, cooperation, friendship, honesty, determination, and integrity (p.109).

Although VBE can play an important role in safeguarding the Spirit of Sport and promoting clean sport values, across athlete age groups and types of sport, research has shown that this process is not straightforward. Specifically, values represent mental representations stored in memory and, as such, their priority are subject to change, such as cognitive re-structuring or reappraisal which, in turn, serves to justify moral transgressions (e.g., Maftei et al., 2019).

The question, however, arises whether the Spirit of Sport values endorsed by WADA as the guiding principles of sport involvement are truly universal to all athletes around the world, governed by individual sport governing bodies, national anti-doping organizations, and perhaps most powerfully by their cultural expectations and general values. Therefore, it is important to further examine if general values and Spirit of Sport values are associated with clean sport values, and to establish if further cultural differences exist between athletes' values. If differences existed, VBE would need to consider these in both the development and implementation of the intervention strategy. Research therefore is needed to determine whether values identified in athletes vary in culture.

## The Present Study

Thus, the current research aims to investigate the importance of general values and those that underpin the Spirit of Sport to athletes and analyze whether any cross-country differences exist in this importance of values. This will inform the further development and implementation of VBE through an understanding of cultural differences and how VBE should be tailored to different audiences. To investigate whether cultural differences exist, the following research questions guided the design of the current study:

1. Are there any differences in the perceived importance of clean sport between athletes of different countries?
2. Are there any differences in the relative importance of general values between athletes of different countries?
3. Are there any differences in the relative importance of Spirit of Sport values between athletes of different countries?
4. Which Spirit of Sport values correlate with perceived importance of clean sport and are there cross-country differences?

## Method

### Participants

Following the receipt of institutional ethical approval (ER21157484), participants were recruited through personal contact networks of the researchers, through university networks and online recruitment platforms (i.e., Prolific), universities (for participants who are 18 years old or older), sport clubs (covering the same age range), and relevant sport organizations/charities. The survey was a cross-sectional single time-point investigation.

Participants were recruited from five European countries (i.e., Germany, Greece, Italy, Russia, and the UK). These countries were selected due to their location in different parts of Europe (e.g., east, west, north, and south) and because they represent distinct cultures [see Hofstede (2011)]. Hofstede's cultural dimensions were supplemented with an additional, tight vs. loose culture, dimension (Gelfand et al., 2011). Four of the five countries were represented in Gelfand and colleagues' research. Therefore, the culture survey data for Russian participants were normalized and displayed relative to the other countries (Appendix 1). The country profiles are included in the Appendix. Based on the existing cultural differences between the participating countries, we expected that comparing these countries is likely to establish any cultural differences between athletes based on their values.

A total of 1225 individuals participated in the study with an age range between 14 and 60 years (*mean age* = 21.50; *SD* = 4.57; males = 618, females = 580, other = 27). These participants were recruited from Germany, Greece, Italy, Russia, and the UK (see Table 1 for the breakdown of participants for each country). The respondents to the survey were active local competitive athletes (*N* = *364*), active national/international competitive athletes (*N* = 290), recreational exercisers (*N* = 439), former athletes (*N* = 82), and those who had no sport involvement (*N* = 39; *N* = 11 missing values). In total, 38 different sports were represented by the participant sample including team sports (i.e., American football, soccer, volleyball) and individual sports (i.e., boxing, weightlifting, running). For the purpose of analysis, the respondents who were recreational exercisers, former athletes, and who had no sport involvement were excluded from further analysis as they are unlikely to possess knowledge of the anti-doping environment or experience VBE. No information on response rate is available because participation was voluntary and no personal links to the study were distributed.

**Table 1:** Participant characteristics.

	***N***	***M* age**	**Gender**		**Athlete level**	***n***
Total	1,225	21.23	Male	618	Active local athlete	364
			Female	580	Active national/international athlete	290
			Other	27	Recreational exerciser	439
					Former athlete	82
					No sport involvement	39
					Missing	11
Italy	251	21.86	Male	144	Active local athlete	77
			Female	107	Active national/international athlete	22
					Recreational exerciser	101
					Former athlete	48
					No sport involvement	3
Germany	257	21.31	Male	113	Active local athlete	46
			Female	134	Active national/international athlete	75
			Other	10	Recreational exerciser	130
					Former athlete	5
					Missing	1
Greece	198	20.33	Male	95	Active local athlete	66
			Female	89	Active national/international athlete	26
			Other	14	Recreational exerciser	74
					No sport involvement	30
					Missing	2
Russia	272	20.11	Male	163	Active local athlete	89
			Female	108	Active national/international athlete	118
			Other	1	Recreational exerciser	64
					Missing	1
UK	247	23.79	Male	103	Active local athlete	86
			Female	142	Active national/international athlete	49
			Other	2	Recreational exerciser	70
					Former athlete	29
					No sport involvement	6
					Missing	7

The remaining sample consisted of a total of 654 (*mean age* = 20.47; SD = 3.75; males = 354, females = 292, other = 8) active local competitive athletes (*N* = 364) and active national/international competitive athletes (*N* = 290). *Distribution* analysis of the remaining sample identified that there were no statistically significant differences between the five countries for competitive level, age, and gender (*p* > 0.05). This means that the samples are similar and can be analyzed and discussed against each other (Table 2).

**Table 2:** Breakdown of final sample.

**Country**	***N***	**Gender**			**Sport level**	
		**Male**	**Female**	**Other**	**Active (local) competitive athlete**	**Active (national/international) competitive athlete**
Overall	654	354	292	8	364	290
Germany	121	57	60	4	46	75
Greece	92	56	32	4	66	26
Italy	99	68	31		77	22
Russia	207	118	89		89	118
UK	135	55	80		86	49

### Instruments

#### Demographics

Respondents were asked to respond to items relating to participant country of nationality, gender, age, sport, and competitive level (active competitive athlete, active amateur athlete, recreational exerciser, former athlete, or no sport involvement).

#### Importance of Clean Sport

To measure importance of clean sport, participants were asked to rate how important clean sport was to them on a 5-point Likert-type scale (−2 to 2): How important is clean sport to you? with rating scale of “*not important at all*” (−2), “*neither important or unimportant*” (0), and “*very important*” (2).

#### Spirit of Sport and General Values

A Balanced Incomplete Block Design MaxDiff (Bose, 1939; Louviere et al., 2013; Best-Worst Scaling) method was used to individually establish the relative importance of the values which underpin Schwartz's 10 general values (1992; Appendix 3) and the 11 Spirit of Sport values (Appendix 3; WADA, 2021a). In the latest iteration of the WADA code (2021a), a 12th item (“athletes' rights as set forth in the Code”) has been added; however, this differs from the Olympic-ideal driven values which were examined in the current study. Ten blocks of five values were presented for general values eleven blocks of five values were presented for the Spirit of Sport values. Each value was presented in five blocks and the order in which they appeared within the blocks and the order to the blocks themselves were randomized to preclude any order effects. Participants were asked to rate which one of five presented values were of most importance and of least importance to them.

#### Translation of Scales

The Spirit of Sport values in German and Russian were not available and were therefore translated via the translation–back translation method (Brislin, 1970). See Appendix 6 for the German and Russian translations.

### Analysis

Data were entered into SPSS 26.0 and checked for any missing values. Initial ANOVA and correlation analysis was conducted. The open-source R program and the support BWS package were used to conduct the MaxDiff (Best Worst Scaling) analysis. This used a multinomial logit (MNL) model approach to analyzing group differences. Fisher Z-transformation was conducted to transform the sampling distribution of Pearson's *r*. This was conducted to test the significance of the difference between two correlation coefficients.

## Results

### Importance of Clean Sport

ANOVA identified significant univariate effects of participant nationality [*F*_(4, 1,204)_ = 797.060, *p* < 0.000) on the perceived importance of clean sport to the respondent. *Post-hoc* Bonferroni tests for participant nationality revealed that athletes from Italy (*M* = 4.89, *SD* = 0.37) and the UK (*M* = 4.66, *SD* = 0.74) placed a significantly higher importance (*p* < 0.001) on clean sport than those from Germany (*M* = 2.73, *SD* = 1.58), Greece (*M* = 1.60, *SD* = 0.64), and Russia (*M* = 1.17, *SD* = 0.90). There were additional significant differences between German, Greek, and Russian participants. All countries other than the UK and Italy significantly differed from each other. There were no significant differences identified for participant gender on the importance placed on clean sport.

### General Values

Using a counts approach for the full sample, *enjoying life and doing things that give pleasure* was computed as being the most important guiding principle in the participants' lives and *doing things in traditional ways to keep up customs* was the least important (see Table 3 for the full rankings).

**Table 3:** Count analysis rankings for general value statements in entire sample.

**Value**	**Rank (count)**
Enjoying life and doing things that give pleasure	1
Every person in the world should be treated equally	2
Helping and responding to the needs of others	3
Taking risks and trying new things	4
Doing things in their own way	5
Having things organized, clean and stable	6
Behaving properly to avoid doing anything wrong	7
Being successful and doing better than others	8
Being the leader and the one making decisions	9
Doing things in traditional ways to keep up customs	10

**Figure 1:**
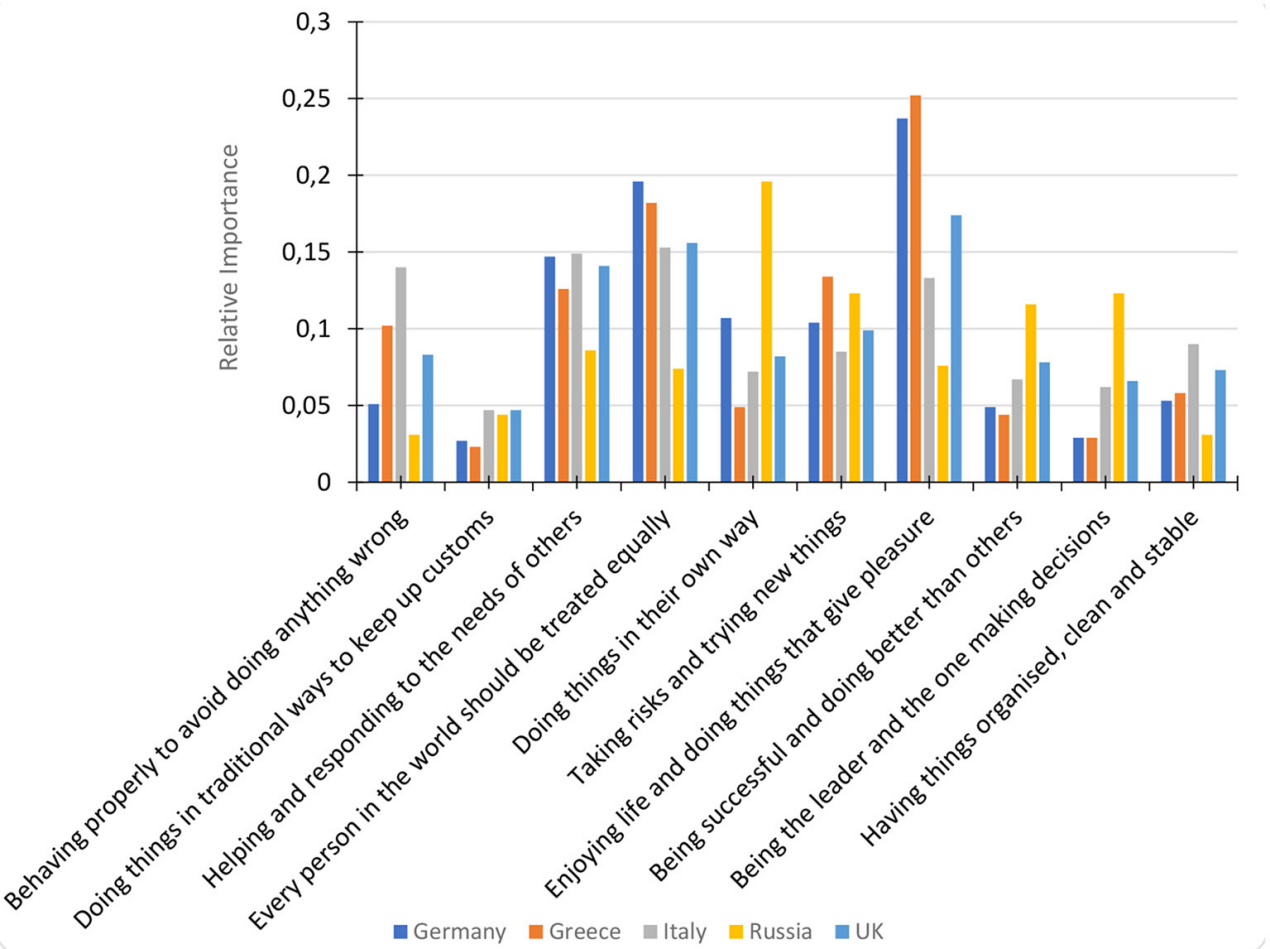
MaxDiff relative importance of general values by participant nationality.

Using a multinomial logit (MNL) model, the relative importance of the 10 general value statements was determined by calculating the share of preferences each value was attributed by participants. Further MaxDiff analysis identified that there were significant differences in the relative importance of general values by participant country (*p* < 0.05). For German, Greek, and UK participants, the value with the highest relative importance was *Enjoying life and doing things that give pleasure*; however, Russian participants reported that *Doing things in their own way* had the highest relative importance and *Every person in the world should be treated equally* was most important for Italian participants (see Figure 1).

### Spirit of Sport Values

Using the counts approach for the full sample, *respect for self and other participants* was computed as being the most important Spirit of Sport value and *displaying courage* was the least important (see Table 4 for the full rankings).

**Table 4:** Counts analysis rankings for spirit of sport statements for entire sample.

	**Rank (count)**
Respect for self and other participants	1
Fun and joy	2
Playing fairly with honesty and ethics	3
Health is an important value of sport	4
Respecting the rules and laws of sport	5
Community and solidarity	6
Dedication and commitment	7
Character and education	8
Working as part of a team	9
Excellence in performance	10
Displaying courage	11

**Figure 2:**
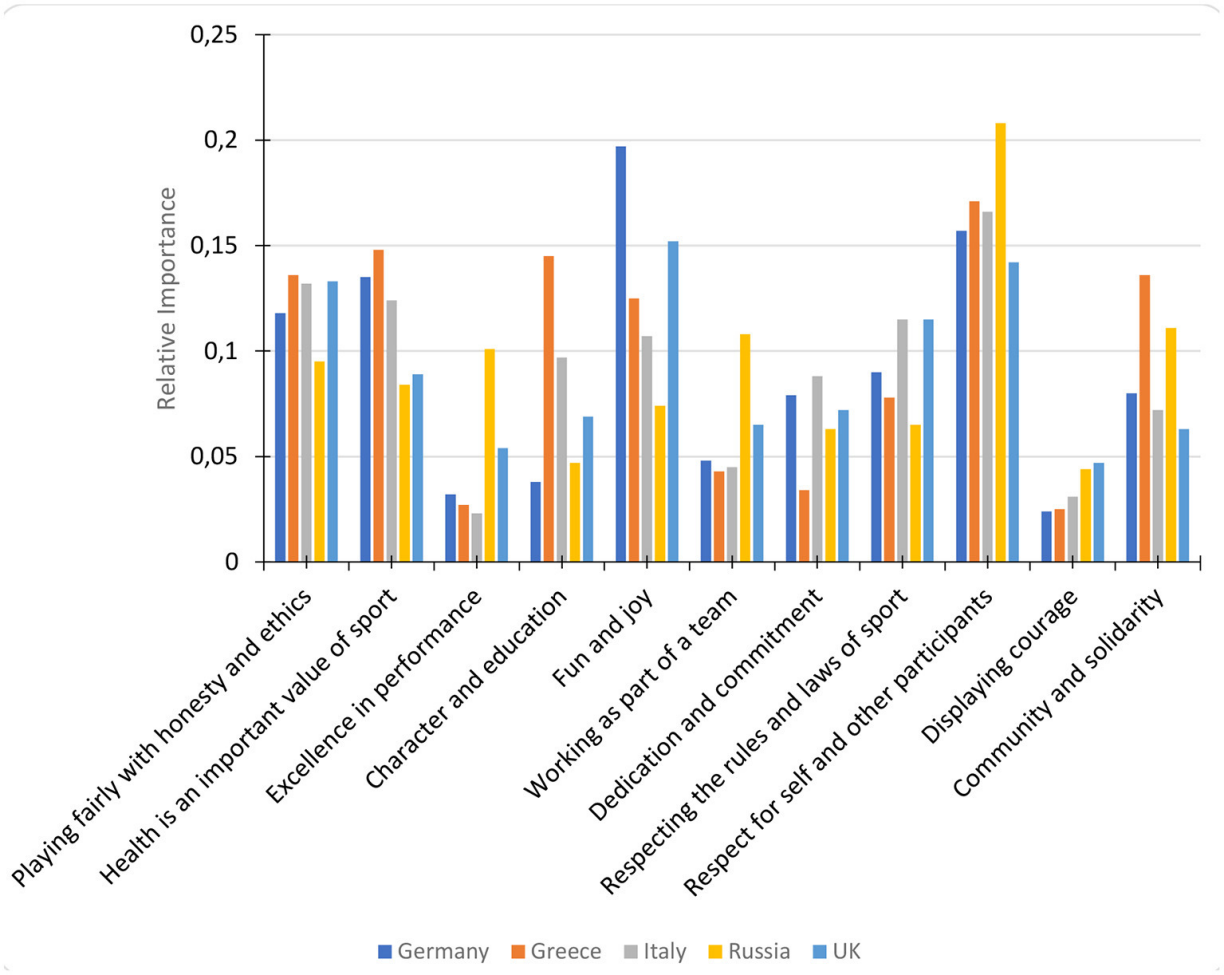
MaxDiff relative importance of Spirit of Sport values by participant nationality.

Using a multinomial logit (MNL) model, the relative importance of the 11 Spirit of Sport value statements was determined by calculating the share of preferences each value was attributed by participants. Further analysis identified that there were significant differences in the relative importance of Spirit of Sport values by participant nationality. In particular, the relative importance of *Community and Solidarity* and *Excellence in Performance* were high for Russian participants, with low relative importance for participants from the other four countries (see Figure 2).

#### Spirit of Sport and the Importance of Clean Sport

A Pearson correlation was run to determine the relationship between perceived importance of clean sport and the 11 underpinning values of the Spirit of Sport. There was a moderate to large, positive correlation (Cohen, 1992) between importance of clean sport and *honesty and ethics* (*r* = 0.538, *p* < 0.005) and *respecting rules and laws* (*r* = 0.507, *p* < 0.005). There were weak, positive correlations between importance of clean sport and *health* (*r* = 0.454, *p* < 0.005), *character and education* (*r* = 0.437, *p* < 0.005), *fun and joy* (*r* = 0.473, *p* < 0.005), *dedication and commitment* (*r* = 0.433, *p* < 0.005), and *respect for self and others* (*r* = 0.484, *p* < 0.005).

There were significant differences in the correlations between importance of sport and the Spirit of Sport values for the respective countries (Table 5) and the total number of significant differences, along with the countries which differ and the Spirit of Sport value they differ on. These are presented in Table 6. Due to the number of significant differences between country correlations, and for brevity, the *z* values where significant differences exist are presented in the Appendix. In total, there are more significant differences than commonalities among which values relate to clean sport within the five countries.

**Table 5:** Individual country correlations between importance of clean sport and spirit of sport values.

	**Honesty and ethics**	**Health**	**Excellence in performance**	**Character and education**	**Fun and joy**	**Teamwork**	**Dedication and commitment**	**Respecting rules and laws**	**Respect for self and others**	**Courage**	**Community and solidarity**
Germany	0.254	−0.035	−0.153	−0.144	−0.145	−0.008	−0.067	0.185	0.039	−0.214	−0.003
Greece	0.471	0.175	−0.034	0.271	−0.099	0.374	0.093	0.356	0.291	−0.013	0.365
Italy	−0.047	0.242	−0.071	0.163	−0.056	−0.148	−0.018	−0.029	−0.029	0.074	0.118
Russia	0.025	0.177	0.044	−0.046	−0.037	0.037	−0.218	0.194	−0.002	0.052	−0.016
UK	0.176	0.002	−0.074	0.004	−0.025	0.025	0.063	0.072	0.073	−0.193	−0.054

**Table 6:** Significant differences between country correlations of importance of clean sport and spirit of sport values.

	**Germany**	**Greece**	**Italy**	**Russia**	**UK**
Germany		1, 4, 5, 6, 9, 11	1, 2, 4, 10	1, 2, 3, 10	–
Greece	1, 4, 5, 6, 9, 11		1, 6, 8, 9, 11	1, 4, 6, 7, 9, 11	1, 4, 6, 8, 9, 11
Italy	1, 2, 4, 10	1, 6, 8, 9, 11		4, 7, 8	1, 2, 10
Russia	1, 2, 3, 10	1, 4, 6, 7, 9, 11	4, 7, 8		7, 10
UK	–	1, 4, 6, 8, 9, 11	1, 2, 10	7, 10	

*1, Honesty and ethics; 2, Health; 3, Excellence in performance; 4, Character and education; 5, Fun and joy; 6, Teamwork; 7, Dedication and commitment; 8, Respecting rules and laws; 9, Respect for self and others; 10, Courage; 11, Community and solidarity; all interaction significant to p < 0.05*.

The largest number of significant differences observed between countries was six values (Germany and Greece; Greece and Russia; Greece and UK). Greece and Italy significantly differed on five values and Germany significantly differed on four values with both Italy and Russia. The UK and Germany did not differ significantly on any of the 11 Spirit of Sport values. The most significant differences observed by country were for participants from Greece with 23 significant differences compared with Germany (16), Italy (15), Russia (17), and the UK (11).

## Discussion

The importance of VBE as a tool within the anti-doping education to prevent and protect athletes from doping has previously been highlighted both in research (cf., Petróczi et al., 2017) and by the new WADA International Standard for Education (ISE; WADA, 2021a). However, the extent to which this VBE can be prescribed to all athletes in different countries, from different cultures and backgrounds, who experience different daily experiences and pressures, is questionable. This assertion does not impact the potential benefits of VBE but highlights the need for a rigorous and flexible development and implementation. To surpass this goal and to better the provision of anti-doping education for athletes, it is important to understand how these individuals from different countries prioritize their values, both in general, everyday life and within their sporting world. It is also critical to understand the impact that the values which underpin the Spirit of Sport (WADA, 2021a) have on an athletes' beliefs of the importance of clean sport and their contribution to maintaining clean sport (Mortimer et al., 2020).

### Values and the Importance of Clean Sport

The first research question related to the difference in perceived importance of clean sport between the five participant nationalities. The analysis revealed significant differences between Italy and the UK and the remaining three countries. This indicates that for those participants from Italy and the UK, clean sport, and the ideals that this represents, is of high importance and a key consideration when preparing and competing in sport, in addition to when any considerations toward doping are made. This does not preclude those participants from Germany, Greece, and Russia from believing in the importance of clean sport; however, it is of less importance and perhaps less of an anti-doping preventative barrier to these athletes.

In relation to the general values which act as underpinning principles in ones' life, enjoying life and doing things that give pleasure was the most important to German, Greek, and UK participants; in addition, German and UK participants reported the importance of fun and joy in sport, highlighting that in both daily life and sport enjoyment is the key underpinning value that drives involvement in activities. Russian athletes, however, were guided by doing things in their own way and a belief of equality was the most important for Italians. The importance for Russians to create their own path and be witnessed to be doing so can be linked to the high relative importance of achieving excellence in performance. However, it is interesting that a general value associated with individualism was reported as important by participants who also rated community and solidarity as important values within sport.

Unsurprisingly, there were positive correlations between the majority of the Spirit of Sport values and the respondents' perceived importance of clean sport. In particular, the moderate positive correlations observed with the values of honesty and ethics and respecting the rules and laws of sport indicate that when an athlete's behavior is guided by these values, they are more likely to believe in the importance of clean sport, and therefore are less likely to engage in doping behaviors, mirroring the findings of Mazanov et al. (2019) that suggest that these values are associated with clean sport likelihood. These relationships are important to consider when developing and implementing VBE as targeting these values, and therefore athlete perceptions of the importance of clean sport may provide an avenue by which to protect and prevent individuals from doping.

### Relative Importance of the Spirit of Sport Values

The present findings confirm previous evidence on the universal nature of the values of sport (Mazanov et al., 2019). Across the five countries, the top two values were “respect for self and others” (also shared by athletes in all countries but in the UK) and “fun and joy” (shared only by German and UK athletes). Values ranked the lowest were “excellence in performance” (shared by athletes in all countries but Russia) and “courage” (universally agreed among all five countries). Our results broadly align with previously recorded rankings of the Spirit of Sport values in Australia, Greece (Mazanov et al., 2019), and in the UK (Mortimer et al., 2020). Courage and excellence in performance appear to be universally unimportant, whereas respect and health valued the most—at least in the countries investigated to date. More specifically, this evidence suggests that although a consistent pattern of values exists, not all sport values are equally important across all countries and nations. While country differences in values' relative importance have been detected, this finding is likely to be related to the dynamic changes in values' priorities at the individual level (Mazanov et al., 2012). Respect for self and other participants seem to have been consistently rated as important sport values. On the other hand, ethics, fair play and honesty, and health, the two main pillars of anti-doping education, have been rated as having lower importance and differences across the participating countries emerged. These findings further corroborate Mazanov et al.'s (2019) conclusion that the moral component of anti-doping is less universal than considered by anti-doping authorities.

The most recent iteration of the WADA Code (2021a) introduced a 12th item to the Spirit of Sport entitled “athletes' rights as set forth in the Code”; however, this item does not align with the previous Olympic-ideal driven values. This highlights the consideration that the values of sport are not a timeless, universal set of values that are appropriate to everyone but a living set of values that change in response to the changing demands in the environment.

### Relationship Between General Values and the Spirit of Sport

This study also sought to investigate these important relationships between general values, Spirit of Sport values, and the importance of clean sport, and how these differ across five European countries. Although guidelines for ISE clearly recommend VBE to be tailored to local context and local values, the clean sport environment for athletes competing internationally is global. Therefore, the global context of elite sport should be considered. The situations and challenges which arise on the global stage may differ significantly from those experienced locally. In turn, the values and value priorities incubated at a local level which have maintained an adherence to clean sport should be nurtured and developed to be able to combat these foreign actors. In addition, the extent to which values are shared between countries and localities and how these values are related to an individual's perception of the importance of clean sport can aid in the development of effective VBE.

## Limitations and Future Research

While the current study identifies cross-country differences, it does not examine how individuals' value-priorities change (on a situation/daily basis). Therefore, we believe that the findings of this study should act as a catalyst for further investigations into how values and value-priorities work at the individual level. In addition, the contrast between value-priorities at the local/country level and the need for athletes to compete on a global stage creates further avenues for further investigation. This research should identify the impacts of the possible tension caused by global competition, and the rewards that come with it, and individual clean sport behavior (and its underlying values). Future research should also investigate whether age differences exist with regards to value priorities which can be fluid and dependent on situational context.

David Howman, then Director General of WADA, said in 2015 at the International Athlete Forum for 2020: “…*it does not matter if you are Director General of WADA, or if you're an athlete, sports fan or lawyer, values remain vital if clean, honest sport is to continue to prosper. Our job in the anti-doping community is to ensure that these values are maintained, and the integrity of sport is protected*” (WADA, 2015). Five years on, research into how these vital values can and are operationalized in daily practices is still lacking. While our study answered some questions about cross-cultural relevance of general values and the Spirit of Sport, we identified further issues to explore and raised more questions to answer. Future research is called for to explore the link between the clean sport concept (i.e., the meaning and importance of clean sport to athletes), clean sport behavior, and Spirit of Sport. Based on the observed importance and unimportance of the sport-related values, we hypothesize that clean sport behavior among competitive athletes is likely to be caused by the close alignment of general values preventing cheating in sport with the moral values among the Spirit of Sport values, and the importance of health and respect for the self being the driving forces in recreational athletes. The consistent low ranking of “excellence is performance” is a perplexing outcome which warrants further exploration. From the research point of view, values in the middle ranks also present an intriguing question, namely if the reason for a more neutral ranking is caused by indifference, or more likely, large disagreement at the individual level as observed previously with the BWS method (Bleasdale et al., 2018).

Recently, Barkoukis and Elbe (2021) suggested that more research is needed to identify whether doping is a moral or an ethical issue and accordingly inform anti-doping education. In support of this argument, our findings show that people in different parts of the world attach different importance to different sport values. These differences may be ascribed to historical, cultural, and societal reasons and suggest that a one size fits all VBE could not serve well in the fight against doping. Future development of VBE should therefore place a stronger focus on designing the interventions in a culturally relevant manner. This implies that when developing VBE materials and deciding on the mode of delivery, the first step needs to be to assess athletes' values in a culturally specific manner to identify which values are related to importance of clean sport. Only after this step has occurred should specific VBE content be developed and delivered.

## Conclusion

The findings of this research study indicate that there are differences in the underpinning principles that guide athletes' behavior both in daily life and within their sporting worlds. This has important value to the field of VBE as it highlights the need to better tailor anti-doping interventions to the cultural backgrounds and pre-defining characteristics of an athlete. Future research and VBE work should endeavor to consider the findings of this study to better protect and prevent athletes from engaging in doping behaviors.

## Data Availability Statement

The raw data supporting the conclusions of this article will be made available by the authors, without undue reservation.

## Ethics Statement

The studies involving human participants were reviewed and approved by Sheffield Hallam University. Written informed consent from the participants' legal guardian/next of kin was not required to participate in this study in accordance with the national legislation and the institutional requirements.

## Author Contributions

TW collected data, analyzed the data, and wrote up the manuscript. AP designed the study and contributed to the data collection, data analysis, and writing of the manuscript. All authors contributed to the article and approved the submitted version.

## Conflict of Interest

The authors declare that the research was conducted in the absence of any commercial or financial relationships that could be construed as a potential conflict of interest.
