# Supplementary material for: One Does Not Fit All: European Study Shows Significant Differences in Value-Priorities in Clean Sport
Source: Front Sports Act Living. 2021 May 24;3:662542. doi: 10.3389/fspor.2021.662542 (PMC8181163; doi:10.3389/fspor.2021.662542)
Supplement: Supplementary file 1 [file Data_Sheet_1.docx]

**Appendix**

Appendix 1. Hofstede’s national culture dimensions and Gelfand’s tight-loose culture dimension for the five included countries

Appendix 2. Full correlation statistics for Spirit of Sport and Importance of Clean Sport by Country

| Honesty & Ethics |  | Germany | Greece | Italy | Russia | UK |
| --- | --- | --- | --- | --- | --- | --- |
|  | Germany |  | -1.73 | 2.22 | 2.00 | - |
|  | Greece | -1.78 |  | 3.80 | 3.82 | - |
|  | Italy | 2.22 | 3.8 |  | - | -1.69 |
|  | Russia | 2 | 3.82 | - |  | - |
|  | UK | - | - | -1.69 | - |  |
|  |  |  |  |  |  |  |
| Health |  | Germany | Greece | Italy | Russia | UK |
|  | Germany |  | - | -2.04 | -1.82 | - |
|  | Greece | - |  | - | - | - |
|  | Italy | -2.04 | - |  | - | 1.85 |
|  | Russia | -1.82 | - | - |  | - |
|  | UK | - | - | 1.85 | - |  |
|  |  |  |  |  |  |  |
| Character & Education |  | Germany | Greece | Italy | Russia | UK |
|  | Germany |  | -2.98 | -2.24 | - | - |
|  | Greece | -2.98 |  | - | 2.55 | 2.01 |
|  | Italy | -2.24 | - |  | 1.70 | - |
|  | Russia | - | 2.55 | 1.70 |  | - |
|  | UK | - | 2.01 | - | - |  |
|  |  |  |  |  |  |  |
| Dedication & Commitment |  | Germany | Greece | Italy | Russia | UK |
|  | Germany |  | - | - | - | - |
|  | Greece | - |  | - | 2.47 | - |
|  | Italy | - | - |  | 1.65 | - |
|  | Russia | - | 2.47 | 1.65 |  | 2.57 |
|  | UK | - | - | - | 2.57 |  |
|  |  |  |  |  |  |  |
| Respecting Rules and Laws |  | Germany | Greece | Italy | Russia | UK |
|  | Germany |  | - | - | - | - |
|  | Greece | - |  | 2.73 | - | 2.20 |
|  | Italy | - | 2.73 |  | -1.83 | - |
|  | Russia | - | - | -1.83 |  | - |
|  | UK | - | 2.20 | - | - |  |
|  |  |  |  |  |  |  |
| Fun and Joy |  | Germany | Greece | Italy | Russia | UK |
|  | Germany |  | -1.73 | - | - | - |
|  | Greece | -1.73 |  | - | - | - |
|  | Italy | - | - |  | - | - |
|  | Russia | - | - | - |  | - |
|  | UK | - | - | - | - |  |
|  |  |  |  |  |  |  |
| Excellence in Performance |  | Germany | Greece | Italy | Russia | UK |
|  | Germany |  | - | - | -1.69 | - |
|  | Greece | - |  | - | - | - |
|  | Italy | - | - |  | - | - |
|  | Russia | -1.69 | - | - |  | - |
|  | UK | - | - | - | - |  |
|  |  |  |  |  |  |  |
| Teamwork |  | Germany | Greece | Italy | Russia | UK |
|  | Germany |  | -2.72 | - | - | - |
|  | Greece | -2.72 |  | 3.69 | 2.80 | 2.70 |
|  | Italy | - | 3.69 |  | - | - |
|  | Russia | - | 2.80 | - |  | - |
|  | UK | - | 2.70 | - | - |  |
|  |  |  |  |  |  |  |
| Respect for Self and Others |  | Germany | Greece | Italy | Russia | UK |
|  | Germany |  | -1.84 | - | - | - |
|  | Greece | -1.84 |  | 2.24 | 2.37 | 1.66 |
|  | Italy | - | 2.24 |  | - | - |
|  | Russia | - | 2.37 | - |  | - |
|  | UK | - | 1.66 | - | - |  |
|  |  |  |  |  |  |  |
| Courage |  | Germany | Greece | Italy | Russia | UK |
|  | Germany |  | - | -2.11 | -2.29 | - |
|  | Greece | - |  | - | - | - |
|  | Italy | -2.11 | - |  | - | 2.03 |
|  | Russia | -2.29 | - | - |  | 2.24 |
|  | UK | - | - | 2.03 | 2.24 |  |
|  |  |  |  |  |  |  |
| Community and Solidarity |  | Germany | Greece | Italy | Russia | UK |
|  | Germany |  | -2.72 | - | - | - |
|  | Greece | -2.72 |  | 1.80 | 3.13 | 3.21 |
|  | Italy | - | 1.80 |  | - | - |
|  | Russia | - | 3.13 | - |  | - |
|  | UK | - | 3.21 | - | - |  |

Appendix 3. Study measures of Schwartz’s Values (1992)

Sets of brief statements describing personal values are presented below. We are interested in your views about how important the values listed below are to you, as guiding principles in your life.

For each set of values below, please choose the ONE value out of those five which, you think, is most important as a guiding principle in your life and choose the ONE value out of those five which, you think, is least important as a guiding principle in your life.

There is NO right answer, the statements repeat, do not think too much, answer quickly – first reactions best capture your life values.

Example: For a soft drink, which attribute is the most and the least important?

| ***Least Important***  *Select ONE* |  | ***Most Important***  *Select ONE* |
| --- | --- | --- |
|  | *Taste* | ***✓*** |
|  | *Sugar content* |  |
|  | *Brand* |  |
| ***✓*** | *Price* |  |
|  | *Free of artificial colouring* |  |

| **Least Important**  Select ONE |  | **Most Important**  Select ONE |
| --- | --- | --- |
|  | Enjoying life and doing things that give pleasure |  |
|  | To be the leader and the one making decisions |  |
|  | Having things organized, clean and stable |  |
|  | Behaving properly to avoid doing anything wrong |  |
|  | To do things in your own way |  |
|  |  |  |
| **Least Important**  Select ONE |  | **Most Important**  Select ONE |
|  | Helping and responding to the needs of others |  |
|  | Every person in the world should be treated equally |  |
|  | To be the leader and the one making decisions |  |
|  | Behaving properly to avoid doing anything wrong |  |
|  | To do things in your own way |  |
|  |  |  |
| **Least Important**  Select ONE |  | **Most Important**  Select ONE |
|  | To do things in your own way |  |
|  | Helping and responding to the needs of others |  |
|  | Taking risks and trying new things |  |
|  | To do things in traditional ways to maintain customs |  |
|  | Having things organized, clean and stable |  |
| **Least Important**  Select ONE |  | **Most Important**  Select ONE |
|  | Behaving properly to avoid doing anything wrong |  |
|  | Every person in the world should be treated equally |  |
|  | Enjoying life and doing things that give pleasure |  |
|  | To do things in traditional ways to maintain customs |  |
|  | Taking risks and trying new things |  |
| **Least Important**  Select ONE |  | **Most Important**  Select ONE |
|  | Enjoying life and doing things that give pleasure |  |
|  | Having things organized, clean and stable |  |
|  | To be the leader and the one making decisions |  |
|  | Every person in the world should be treated equally |  |
|  | Taking risks and trying new things |  |
|  |  |  |
| **Least Important**  Select ONE |  | **Most Important**  Select ONE |
|  | To be the leader and the one making decisions |  |
|  | Helping and responding to the needs of others |  |
|  | Enjoying life and doing things that give pleasure |  |
|  | To do things in traditional ways to maintain customs |  |
|  | Being successful and doing better than others |  |
|  |  |  |
| **Least Important**  Select ONE |  | **Most Important**  Select ONE |
|  | Taking risks and trying new things |  |
|  | Helping and responding to the needs of others |  |
|  | Behaving properly to avoid doing anything wrong |  |
|  | Having things organized, clean and stable |  |
|  | Being successful and doing better than others |  |
|  |  |  |
| **Least Important**  Select ONE |  | **Most Important**  Select ONE |
|  | To be the leader and the one making decisions |  |
|  | To do things in your own way |  |
|  | Taking risks and trying new things |  |
|  | To do things in traditional ways to maintain customs |  |
|  | Being successful and doing better than others |  |
|  |  |  |
| **Least Important**  Select ONE |  | **Most Important**  Select ONE |
|  | Helping and responding to the needs of others |  |
|  | Being successful and doing better than others |  |
|  | Enjoying life and doing things that give pleasure |  |
|  | To do things in your own way |  |
|  | Every person in the world should be treated equally |  |
|  |  |  |
| **Least Important**  Select ONE |  | **Most Important**  Select ONE |
|  | Being successful and doing better than others |  |
|  | Every person in the world should be treated equally |  |
|  | Behaving properly to avoid doing anything wrong |  |
|  | Having things organized, clean and stable |  |
|  | To do things in traditional ways to maintain customs |  |

**Please use the scale to rate how important each of the following values is to you, as a guiding principle in your life.**

| *Opposed to my values* | *Not important* |  |  | *Important* |  |  | *Very important* | *Of supreme importance* |
| --- | --- | --- | --- | --- | --- | --- | --- | --- |
| -1 | 0 | 1 | 2 | 3 | 4 | 5 | 6 | 7 |

**Mark the level of your agreement for each statement:**

| Behaving properly to avoid doing anything wrong. | -1 | 0 | 1 | 2 | 3 | 4 | 5 | 6 | 7 |
| --- | --- | --- | --- | --- | --- | --- | --- | --- | --- |
| Doing things in traditional ways to maintain customs. | -1 | 0 | 1 | 2 | 3 | 4 | 5 | 6 | 7 |
| Helping and responding to the needs of others. | -1 | 0 | 1 | 2 | 3 | 4 | 5 | 6 | 7 |
| Every person in the world should be treated equally. | -1 | 0 | 1 | 2 | 3 | 4 | 5 | 6 | 7 |
| Doing things in your own way. | -1 | 0 | 1 | 2 | 3 | 4 | 5 | 6 | 7 |
| Taking risks and trying new things. | -1 | 0 | 1 | 2 | 3 | 4 | 5 | 6 | 7 |
| Enjoying life and doing things that give pleasure. | -1 | 0 | 1 | 2 | 3 | 4 | 5 | 6 | 7 |
| Being successful and doing better than others. | -1 | 0 | 1 | 2 | 3 | 4 | 5 | 6 | 7 |
| Being the leader and the one making decisions. | -1 | 0 | 1 | 2 | 3 | 4 | 5 | 6 | 7 |
| Having things organized, clean and stable. | -1 | 0 | 1 | 2 | 3 | 4 | 5 | 6 | 7 |

Appendix 4. Study measures for Spirit of Sport Values (WADA, 2021a)

**The “Spirit of Sport” is what the Olympic movement says makes sport intrinsically valuable. We are interested how important the different values that contribute to the Olympic Movement’s “Spirit of Sport” are to you.**

For each set of values below, please choose the **ONE** value out of those five which, you think, is most important to you, and choose the **ONE** value out of those five which, you think, is least important to the Spirit of Sport.

| **Least Important**  Select ONE |  | **Most Important**  Select ONE |
| --- | --- | --- |
|  | Having fun and joy in sport |  |
|  | Showing respect for myself and other participants |  |
|  | Working as part of a team |  |
|  | Respecting the rules and laws of sport |  |
|  | Showing dedication and commitment |  |
|  |  |  |

| **Least Important**  Select ONE |  | **Most Important**  Select ONE |
| --- | --- | --- |
|  | Playing fairly with honesty and ethics |  |
|  | Showing community and solidarity |  |
|  | Working as part of a team |  |
|  | To me, being healthy is important |  |
|  | Showing dedication and commitment |  |
|  |  |  |
| **Least Important**  Select ONE |  | **Most Important**  Select ONE |
|  | Working as part of a team |  |
|  | Having fun and joy in sport |  |
|  | Displaying courage is important to me |  |
|  | Showing community and solidarity |  |
|  | Character and education are important to me |  |
|  |  |  |
| **Least Important**  Select ONE |  | **Most Important**  Select ONE |
|  | To me, being healthy is important |  |
|  | Respecting the rules and laws of sport |  |
|  | Character and education are important to me |  |
|  | Working as part of a team |  |
|  | Excellence in performance is important to me |  |
|  |  |  |
| **Least Important**  Select ONE |  | **Most Important**  Select ONE |
|  | To me, being healthy is important |  |
|  | Having fun and joy in sport |  |
|  | Showing dedication and commitment |  |
|  | Displaying courage is important to me |  |
|  | Excellence in performance is important to me |  |
|  |  |  |
| **Least Important**  Select ONE |  | **Most Important**  Select ONE |
|  | Having fun and joy in sport |  |
|  | Showing respect for myself and other participants |  |
|  | Playing fairly with honesty and ethics |  |
|  | Character and education are important to me |  |
|  | To me, being healthy is important |  |
|  |  |  |
| **Least Important**  Select ONE |  | **Most Important**  Select ONE |
|  | Character and education are important to me |  |
|  | Showing respect for myself and other participants |  |
|  | Showing community and solidarity |  |
|  | Excellence in performance is important to me |  |
|  | Showing dedication and commitment |  |

| **Least Important**  Select ONE |  | **Most Important**  Select ONE |
| --- | --- | --- |
|  | To me, being healthy is important |  |
|  | Showing community and solidarity |  |
|  | Displaying courage is important to me |  |
|  | Showing respect for myself and other participants |  |
|  | Respecting the rules and laws of sport |  |
|  |  |  |
| **Least Important**  Select ONE |  | **Most Important**  Select ONE |
|  | Excellence in performance is important to me |  |
|  | Displaying courage is important to me |  |
|  | Working as part of a team |  |
|  | Showing respect for myself and other participants |  |
|  | Playing fairly with honesty and ethics |  |
|  |  |  |
| **Least Important**  Select ONE |  | **Most Important**  Select ONE |
|  | Displaying courage is important to me |  |
|  | Respecting the rules and laws of sport |  |
|  | Showing dedication and commitment |  |
|  | Character and education are important to me |  |
|  | Playing fairly with honesty and ethics |  |
|  |  |  |
| **Least Important**  Select ONE |  | **Most Important**  Select ONE |
|  | Playing fairly with honesty and ethics |  |
|  | Respecting the rules and laws of sport |  |
|  | Having fun and joy in sport |  |
|  | Showing community and solidarity |  |
|  | Excellence in performance is important to me |  |

**Please rate how important each of the following values is to you, as an intrinsic part of the Spirit of Sport.**

| *Opposed to my values* | *Not important* |  |  | *Important* |  |  | *Very important* | *Of supreme importance* |
| --- | --- | --- | --- | --- | --- | --- | --- | --- |
| -1 | 0 | 1 | 2 | 3 | 4 | 5 | 6 | 7 |

**Mark the level of your agreement for each statement:**

| Playing fairly with honesty and ethics | -1 | 0 | 1 | 2 | 3 | 4 | 5 | 6 | 7 |
| --- | --- | --- | --- | --- | --- | --- | --- | --- | --- |
| To me, being healthy is important | -1 | 0 | 1 | 2 | 3 | 4 | 5 | 6 | 7 |
| Excellence in performance is important to me | -1 | 0 | 1 | 2 | 3 | 4 | 5 | 6 | 7 |
| Character and education are important to me | -1 | 0 | 1 | 2 | 3 | 4 | 5 | 6 | 7 |
| Having fun and joy in sport | -1 | 0 | 1 | 2 | 3 | 4 | 5 | 6 | 7 |
| Working as part of a team | -1 | 0 | 1 | 2 | 3 | 4 | 5 | 6 | 7 |
| Showing dedication and commitment | -1 | 0 | 1 | 2 | 3 | 4 | 5 | 6 | 7 |
| Respecting the rules and laws of sport | -1 | 0 | 1 | 2 | 3 | 4 | 5 | 6 | 7 |
| Showing respect for myself and other participants | -1 | 0 | 1 | 2 | 3 | 4 | 5 | 6 | 7 |
| Displaying courage is important to me | -1 | 0 | 1 | 2 | 3 | 4 | 5 | 6 | 7 |
| Showing community and solidarity | -1 | 0 | 1 | 2 | 3 | 4 | 5 | 6 | 7 |

Appendix 5. Gelfand’s tight/loose culture instrument (Gelfand et al., 2011)

Please read each of the following statements and rate the extent to which you agree that the statement reflects the culture of your country on the following scale:

| *Strongly Disagree* | *Disagree* | *Slightly Disagree* | *Slightly Agree* | *Agree* | *Strongly Agree* |
| --- | --- | --- | --- | --- | --- |
| 1 | 2 | 3 | 4 | 5 | 6 |

Mark your level of agreement for each statement:

| In my country, there are many customs and unwritten rules that people are supposed to obey. | 1 | 2 | 3 | 4 | 5 | 6 |
| --- | --- | --- | --- | --- | --- | --- |
| In my country, there are very clear expectations for how people should act in most situations. | 1 | 2 | 3 | 4 | 5 | 6 |
| In my country, people agree upon what behaviors are appropriate versus inappropriate in most situations. | 1 | 2 | 3 | 4 | 5 | 6 |
| In my country, people have a great deal of freedom in deciding how they want to behave in most situations. | 1 | 2 | 3 | 4 | 5 | 6 |
| In my country, if someone acts in an inappropriate way, others will strongly disapprove. | 1 | 2 | 3 | 4 | 5 | 6 |
| People in my country almost always follow the customs and unwritten rules. | 1 | 2 | 3 | 4 | 5 | 6 |

Appendix 6: German and Russian translation of the Spirit of Sport values.

**Bitte bewerte jeden der folgenden Werte dahingehend, wie wichtig jeder einzelne als wesentlicher Bestandteil des „Spirit of Sports“ für dich ist.**

| *EntgegenmeinenWerten* | *Nichtwichtig* |  |  | *Wichtig* |  |  | *Sehrwichtig* | *Von höchster Wichtigkeit* |
| --- | --- | --- | --- | --- | --- | --- | --- | --- |
| -1 | 0 | 1 | 2 | 3 | 4 | 5 | 6 | 7 |

**Gib den Grad deiner Zustimmung für jede Aussage an:**

| Fair spielen mit Ehrlichkeit und Ethik | -1 | 0 | 1 | 2 | 3 | 4 | 5 | 6 | 7 |
| --- | --- | --- | --- | --- | --- | --- | --- | --- | --- |
| Für mich ist wichtig gesund zu sein | -1 | 0 | 1 | 2 | 3 | 4 | 5 | 6 | 7 |
| Hervorragende Leistungen sind mir wichtig | -1 | 0 | 1 | 2 | 3 | 4 | 5 | 6 | 7 |
| Der Charakter und die Bildung sind mir wichtig | -1 | 0 | 1 | 2 | 3 | 4 | 5 | 6 | 7 |
| Spaß und Freude beim Sport haben | -1 | 0 | 1 | 2 | 3 | 4 | 5 | 6 | 7 |
| Als Teil eines Teams arbeiten | -1 | 0 | 1 | 2 | 3 | 4 | 5 | 6 | 7 |
| Hingabe und Einsatzzeigen | -1 | 0 | 1 | 2 | 3 | 4 | 5 | 6 | 7 |
| Die Regeln und Gesetze des Sports respektieren | -1 | 0 | 1 | 2 | 3 | 4 | 5 | 6 | 7 |
| Respekt gegenüber mir und anderen Teilnehmenden zeigen | -1 | 0 | 1 | 2 | 3 | 4 | 5 | 6 | 7 |
| Mut zu zeigen ist mir wichtig | -1 | 0 | 1 | 2 | 3 | 4 | 5 | 6 | 7 |
| Gemeinschaft und Solidaritätzeigen | -1 | 0 | 1 | 2 | 3 | 4 | 5 | 6 | 7 |

*Russian translation of the Spirit of Sport values*

**Пожалуйста, оцените, насколько важно каждое из следующих значений для вас как неотъемлемая часть “духа спорта”.**

| *Противоречит моим ценностям* | *Не важно* |  |  | *Важно* |  |  | *Очень важно* | *Первостепенное значение* |
| --- | --- | --- | --- | --- | --- | --- | --- | --- |
| -1 | 0 | 1 | 2 | 3 | 4 | 5 | 6 | 7 |

**Отметьте свою степень согласия с каждым утруждением:**

| Играть честно с уважением и этичностью | -1 | 0 | 1 | 2 | 3 | 4 | 5 | 6 | 7 |
| --- | --- | --- | --- | --- | --- | --- | --- | --- | --- |
| Для меня важно быть здоровым | -1 | 0 | 1 | 2 | 3 | 4 | 5 | 6 | 7 |
| Совершенство в своем спорте | -1 | 0 | 1 | 2 | 3 | 4 | 5 | 6 | 7 |
| Хорошие манеры и образование важны для меня | -1 | 0 | 1 | 2 | 3 | 4 | 5 | 6 | 7 |
| Получать удовольствие и радость в спорте | -1 | 0 | 1 | 2 | 3 | 4 | 5 | 6 | 7 |
| Работать в команде | -1 | 0 | 1 | 2 | 3 | 4 | 5 | 6 | 7 |
| Демонстрировать самоотверженность и целеустремленность | -1 | 0 | 1 | 2 | 3 | 4 | 5 | 6 | 7 |
| Соблюдать правила спорта | -1 | 0 | 1 | 2 | 3 | 4 | 5 | 6 | 7 |
| Проявлять уважение к себе и другим участникам | -1 | 0 | 1 | 2 | 3 | 4 | 5 | 6 | 7 |
| Для меня важно проявить смелость | -1 | 0 | 1 | 2 | 3 | 4 | 5 | 6 | 7 |
| Проявлять чувство общности и солидарности | -1 | 0 | 1 | 2 | 3 | 4 | 5 | 6 | 7 |
